# Current approaches to vision restoration using optogenetic therapy

**DOI:** 10.3389/fncel.2023.1236826

**Published:** 2023-08-16

**Authors:** Kashish Parnami, Anwesha Bhattacharyya

**Affiliations:** Amity Institute of Neuropsychology and Neurosciences, Amity University, Noida, Uttar Pradesh, India

**Keywords:** vision restoration, primary visual cortex, optogenetic, bipolar cells, retinal ganglion cells

## Abstract

Inherited progressive degeneration of photoreceptors such as retinitis pigmentosa (RP) is the most common cause of blindness leading to severe vision impairment affecting ~1 in 5,000 people worldwide. Although the function and morphology of the photoreceptors get disrupted, there is evidence that the inner retinal neurons such as bipolar cells and the retinal ganglion cells are left intact until later stages. Among several innovative therapeutic options aiming to restore vision, optogenetic therapy can bestow light sensitivity to remaining retinal neurons by ectopic expression of light-sensitive proteins. Since the advent of this technique, a diverse class of opsins (microbial and mammalian opsins), chimeric proteins, ligand-gated ion channels, and switchable opsins have been used to study their potential in vision restoration. These proteins differ in their excitation spectra, response kinetics, and signal amplification cascade. Although most of the studies have reported high fidelity of responses in the retina, only a handful of them have achieved functional vision in the visual cortex. This review is a summary of the visuocortical and behavioral responses after optogenetic treatment of the degenerated retina. This clarifies to what extent improved and meaningful vision can be obtained for therapeutic efficacy and continued clinical progress.

## Introduction

**Figure 1:**
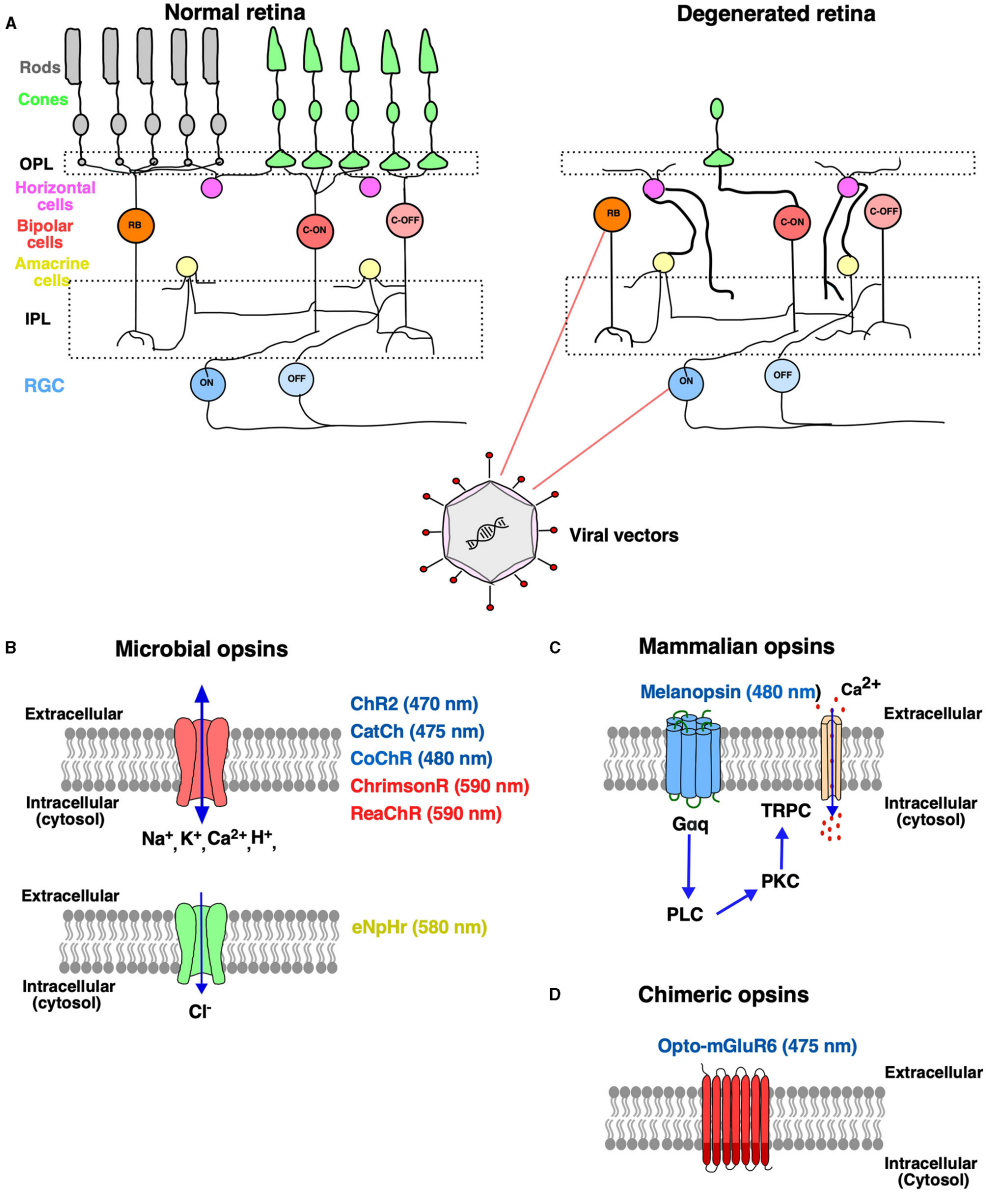
Optogenetic therapy to treat retinal degenerative disease. **(A)** Schematic representation of a normal healthy retina consisting of different types of neurons: photoreceptors (rods and cones), bipolar cells and retinal ganglion cells (RGCs), horizontal cells and amacrine cells. In inherited retinal degeneration, there is a progressive loss of rods and cones, and the retinal circuitry undergoes aberrant changes, while the deeper retinal layers [bipolar cells (BP) and retinal ganglion cells (RGCs)] remain largely intact. Aberrant synaptic connections are formed and represented in bold with infiltration of glial cells. The BP and RGCs have become potential targets for optogenetic therapy turning them into functional photoreceptors. Viral vectors are used for the expression of the opsin gene to the target cell comprising rod and cone bipolar cells and RGCs through intravitreal or subretinal administration **(B–D)**. The optogenetic toolkit comprises of different classes of opsins such as microbial opsins and mammalian opsins. **(B)** The microbial opsins are of two types: depolarizing and hyperpolarizing. Depolarizing opsins (denoted by red color): light-gated ion channels upon light activation allow the entry of cations inside the cell-causing depolarization. Hyperpolarizing opsins (denoted by green color): light-gated chloride pump that inhibits neurons by hyperpolarization in response to yellow or green light stimulation. **(C)** Mammalian opsins, such as melanopsin, a G-protein-coupled receptor (GPCR), which upon light stimulation triggers Gaq activation, followed by phospholipase C (PLC) and protein kinase C (PKC) signaling that further triggers the opening of transient receptor potential ion channel (TRPC), thereby increasing cytosolic Ca^2+^. **(D)** GPCRs have been engineered for enhanced light sensitivity, Opto-mGluR6, which is a chimeric fusion protein composed of the light sensing extracellular and transmembrane domains of melanopsin and the intracellular, G protein coupling domains of the ON-bipolar cell-specific, metabotropic glutamate receptor, mGluR6. OPL, outer plexiform layer; IPL, inner plexiform layer; ChR2, channelrhodopsin; CatCh, calcium-translocating channelrhodopsin; eNpHR, enhanced Natromonas halorhodopsin; CoChR, chloromonas oogama.

The retina is a light-sensitive tissue at the back of the eye consisting of several layers of neurons for processing of light information (photoreceptor-bipolar cells–retinal ganglion cells). When light strikes the retina, the photoreceptors convert the light into an electrical impulse through a cascade of biochemical events. The retinal output is relayed through the optic nerve, optic chiasm, optic tract, dorsal lateral geniculate nucleus, and finally to the visual cortex for image processing and interpretation (Erskine and Herrera, 2014). Retinal degeneration (RD) is the major cause of loss of vision characterized by the progressive degeneration of photoreceptor cells and neuronal remodeling. The most common forms of RD are age-related macular degeneration (AMD) and retinitis pigmentosa (RP) (Katherine et al., 2014). According to reports from the World Health Organization, over 2.2 billion people globally are affected with vision problems, of which 1 billion cases of vision impairment are preventable (Trott et al., 2022). In the past few years, the focus has been to develop innovative experimental therapies for the restoration of naturalistic vision. Among several therapeutic options, optogenetic vision restoration has been successful in restoring vision at the cellular level and has successfully entered clinical trials. The advent of optogenetics has benefitted neuroscience researchers in providing a toolbox that allows optical control of neural circuitry using genetically encoded proteins in target cells (Boyden et al., 2005; Smedemark-Margulies and Trapani, 2013; Sakai et al., 2022). The retina undergoes progressive changes upon the death of the photoreceptors such as sprouting, dendritic retraction of the horizontal cells and bipolar cells, remodeling of neural and glial cells, and the inner nuclear layer changing to a thinner and irregular form (Strettoi et al., 2002). The remnant cones after the loss of outer segments make ectopic synapses with rod bipolar cells (Peng et al., 2000) that form clusters of synchronized spontaneous activity (Haq et al., 2014). A similar increase in spontaneous activity has been shown in the primary visual cortex that results in a lower signal-to-noise ratio and reduced capacity to discriminate stimulus (Wang et al., 2016). Despite the pathologic remodeling in the retina with the onset of photoreceptor loss, the bipolar and retinal ganglion cells (RGCs) survive even in the later stages of inherited RD (Saraf and Olmos de Koo, 2019). The optogenetic strategy of vision restoration offers a unique opportunity to make use of surviving retinal cells by vector-based [adeno-associated virus (AAV) delivery of a transgene encoding a light-sensitive protein (McClements et al., 2020; Lindner et al., 2022; Figure 1). Until now, research has shown successful restoration of light sensitivity at the electrophysiological and behavioral level in animal models of retinitis pigmentosa (RP), a heterogeneous group of retinal disorder resulting in progressive loss of photoreceptor cells leading to impaired vision (Gilhooley et al., 2022; Prosseda et al., 2022).

**Figure 2:**
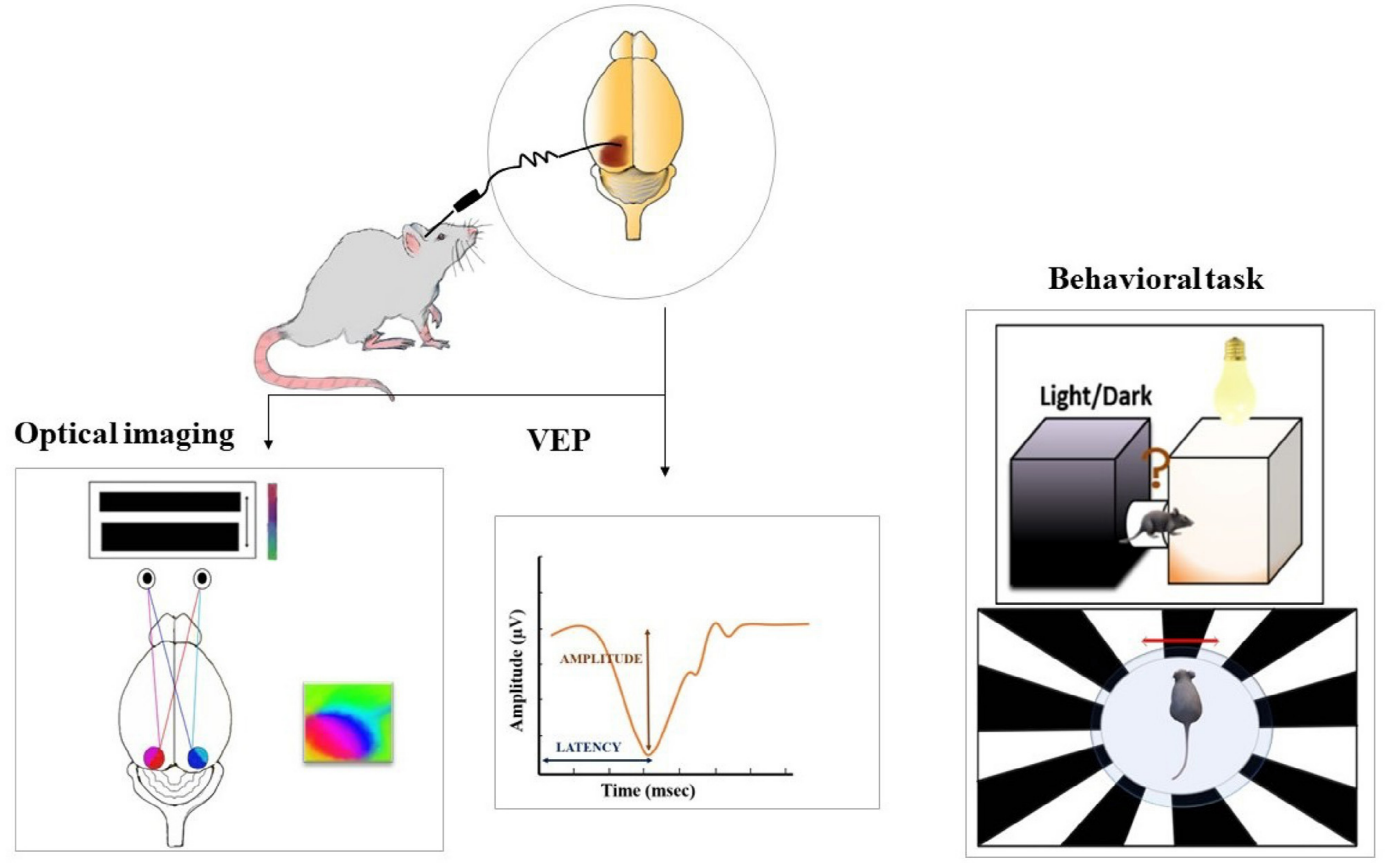
Post-treatment evaluation of the restored vision is assessed using various methods such as optical imaging (measure cortical activity represented by polar maps on the right), electrophysiological recordings (measure the amplitude and latency of visually evoked potential (VEP), and behavioral paradigms. For optical imaging, the intrinsic signal images are acquired as a measure of cortical responses from both hemispheres, while the presentation of continuous visual stimulus to the animal. Visually evoked responses from the visual cortex are evaluated to assess if the transduced retina is able to transmit the information to higher visual centers. The potentials are measured from the entire depth or middle layers of the visual cortex while presentation of a light stimulus to the retina. Light-induced behavioral responses are tested with the help of a light dark box where an increase or decrease in locomotor activity in response to light is measured. Behavioral assessment is also done with an optokinetic drum presenting rotating black and white stripes. This test is performed to measure contrast sensitivity and visual acuity.

The first attempt of inducing light sensitivity in vertebrate neurons was demonstrated by the genetic expression of opsin from the fruit fly retina that elicited action potentials upon illumination (Zemelman et al., 2002). Since then, optogenetics has gained momentum by using microbial (type I) or vertebrate opsins (type II) and converting secondary/tertiary retinal neurons into primary neurons. The initial studies involved viral-mediated expression of channelrhodopsin-2 (ChR2), where ectopic expression of this protein in RGCs and bipolar cells led to light-induced membrane permeability restoring visual function in the rd1 mouse model (Bi et al., 2006; Cehajic-Kapetanovic et al., 2015; Simunovic et al., 2019). The same has also been tested in patients with advanced RP initiated by Retrosense Therapeutics and is currently in Phase I/IIa of the clinical trial (NCT02556736). These studies presented some major challenges such as short wavelength and the requirement of high-intensity light which is toxic for the retina limiting its application in clinical studies. Since then, the focus has been to use human opsins that execute their effect through the native G-protein coupled cascade amplifying the signals and increasing sensitivity. The major disadvantage of this method is the slower kinetics of light response not suited for tracking moving objects (Lin et al., 2008). A recent focus has been more on targeting the bipolar cells compared to retinal ganglion cells to evaluate restored vision where differences in the visual output have been observed (Gilhooley et al., 2021). The visual output is evaluated from visually evoked potentials (VEPs) that have an initial negative deflection, a wave, resulting from hyperpolarization of photoreceptors and a positive deflection, b wave, due to depolarization of ON-bipolar cells (Mahroo, 2023). The amplitude (from negative trough to subsequent positive peak) and latency (time from onset of light stimulation to the negative trough) components of the waves are measured to assess the recovery of vision after therapy. It is important to understand how visual responses differ and to what extent visual acuity can be achieved using optogenetic therapy for maximal translational benefit (Figure 2).

## Visual cortical responses with expression in inner retinal neurons

There are several strategies to impart light sensitivity to the surviving cells of the retina: one is by selective targeted expression of specific cell types and the other is by ubiquitous expression in surviving retinal cells.

To determine the efficacy of optogenetic treatment as a viable option for vision restoration, preliminary studies were conducted by expressing channelrhodopsin-2 (ChR2- Chop2) in inner retinal neurons in *rd1* mice (Bi et al., 2006). The expression profile of the transduced protein was checked by retinal wholemounts and brain sections where RGCs showed predominant expression. The protein expression was stable and helped restore the retina's capacity in encoding light signals and relayed them to the visual cortex. The VEPs had smaller amplitude with shorter latency compared to WT mice, which could be due to lower transfection of the protein in the inner retinal neurons and lack of amplification cascade for signal propagation to higher visual areas. Similar experiments were carried out in another animal model with inherited retinal degenerations, a Royal College of Surgeon (RCS) rat at advanced stages of RD (Tomita et al., 2007). The action of the transgene (AAV-Chop2V) led to low-amplitude VEPs in response to blue light but not to red light stimulation, unlike non-dystrophic rats. Later, the same group tested intravitreal administration with a modified version of the previous transgene, Volvox-channelrhodopsin-1 (mVChR1) having a broader, red-shifted action spectrum suitable for vision restoration (Tomita et al., 2014). The modified vector helped to elicit evoked responses from LED stimulation in longer wavelengths (468–640 nm) which were retained for a year. Ongoing efforts in making channel rhodopsin more light-sensitive led to the development of modified ChR2, ex3mV1CO which is more sensitive than mVChR1 (Watanabe et al., 2021). Blind rats transfected with this gene led to VEP responses at wider wavelengths between 460 and 650 nm at a threshold intensity of 1.0 × 10^11^ photons/cm^2^/s, making it a suitable candidate for daylight vision.

Researchers have demonstrated that ectopic expression of human opsins such as melanopsin (hMel) on the retinal cells can safely restore visual function in blind *rd1* mice at an advanced stage of degeneration (Liu et al., 2016). Although the visual function of the treated mice was restored, the effects were temporary as it was not visible after 45 days of injection. The loss of human melanopsin expression due to species differences could contribute to the loss of visual rescue. Cone photoreceptors also offer the possibility of intervention due to their lifespan. This advantage has offered the benefit of reactivating the cones with light-activated chloride pumps via the AAV virus (Busskamp et al., 2010). The transduced retinas showed sustained, fast, and large cone-driven light responses elicited by reactivated cones to light increments and decrements. In contrast, evoked responses could not be measured from the cortex. The problem with targeting cones is that after the death of rods, the cones start to lose their outer segments leaving a narrow time window to use them for intervention. The progressive degeneration is unlikely to stop even after optogenetic treatment of cones which may lead to improper computation of sensory information. Therefore, targeting cones may not be the best-suited option for patients with end-stage RD where cones are barely left.

Targeting the inner retinal neurons established the proof of concept that optogenetics has the potential to restore visual processing even after retinal degeneration. The drawback in the above studies was that the recovery of vision was mostly suboptimal and not long term. A list of studies in inner retinal neurons is given in Table 1.

**Table 1:** Summary of optogenetic studies targeted on different cells.

**Opsin**	**Cells targeted**	**Promoter/enhancer**	**Transduction method/mode of delivery**	**Animal model and age**	**Results**	**References**
CatCh	Retinal ganglion cells	SNCG (Gamma synuclein), CMV	Intravitreal	Mice (rd1) > 12 weeks, macaque	➢ Increased cortical activity with increasing intensities of blue light. The VEPs had shorter latency and higher amplitude. ➢ Compared to the ubiquitous CMV promoter, SNCG regulates expression in twice as many RGCs.	Chaffiol et al., 2017
Chop2-GFP	Retinal cells	CAG/WPRE	Intravitreal	• Mice (rd1) • P90	➢ VEPs were observed in response to blue light stimulation.	Bi et al., 2006
Chop2	Retinal cells	CAG	Intravitreal	• Rat (RCS) • P300	➢ Blue LED sensitivity was lower in RCS rats. ➢ The VEP amplitude was lower in dystrophic rats compared to non-dystrophic rats.	Tomita et al., 2007
ChR2	ON Bipolar cells (rods and cones)	mGRM6/SV40	Electroporation	• Mice (rd1) • P28	➢ The amplitude of the VEP in treated rd1 mice was greater than in wild-type (WT) mice. ➢ The locomotor activity of the injected mice increased.	Lagali et al., 2008
ChR2	ON Bipolar cells (rods and cones)	mGRM6/SV40	Subretinal	• Mice (rd1, rd10, and rd16) • P56	➢ Improvement in visually directed behavior.	Doroudchi et al., 2011
ChR2	ON Bipolar cells (rods and cones)	mGRM6/SV40	Intravitreal	• Mice (rd1) • P28–P56	➢ The amplitude of VEP was consistently lower in treated mice. Improved light-induced locomotor behavior and restoration of ON and OFF responses at cortical levels.	Macé et al., 2015
ChR2	Retinal ganglion cells	CAG	Intravitreal	• Rat (RCS) • P180	➢ The VEPs had increased amplitude and decreased latency that lasted up to 5 months. ➢ Improvement in behavioral responses	Tomita et al., 2010
ChR2	Retinal ganglion cells	Thy1	ChR2 and rd1 cross (Transgenic mice)	• Mice (rd1) • P100–P250	➢ The mice could reliably discriminate between light and dark fields. ➢ Cortical activation is evoked by the stimulus of high light intensities.	Thyagarajan et al., 2010
ChrimsonR	Foveal Retinal ganglion cells	Ubiquitous promoter	Intravitreal	Macaque (age not mentioned)	➢ The structure of the ganglion cell layer remains preserved 2 years after photoreceptor layer ablation and optogenetic responses were preserved until 1 year.	McGregor et al., 2022
ChrimsonR	Retinal ganglion cells	RGC specific promoter	Intravitreal	Macaque (age not mentioned)	➢ Enhanced capacity of encoding information demonstrated by the ability to decode the direction and speed of moving bars.	Gauvain et al., 2021
ChrimsonR	Retinal ganglion cells	-	Intravitreal	Macaque	➢ VEPs were seen in mice that were given synaptic blockers activated with orange LEDs. Protein expression lasted over 20 months after injection in the perifoveal region of the eye.	Chaffiol et al., 2022
Chrimson R	Retinal ganglion cells	Syn	Intravitreal	• Mice (rd10) • P21	➢ Mice had long-lasting responses to red light.	Cheong et al., 2018
ChrimsonR	Retinal ganglion cells	CAG	Intravitreal	Human	➢ Visual improvement was seen 7 months post-training using light-stimulating goggles. ➢ EEG recordings demonstrated modulation of visual responses in the presence or absence of any object. ➢ Treated patients showed functional recovery of vision being able to recognize objects placed in a closed arena.	Sahel et al., 2021
CoChR	Retinal ganglion cells	CAG	Intravitreal	• Triple knockout (TKO) mice model • P30	➢ TKO mice had enhanced visual functions such as light sensitivity, contrast sensitivity, and visual acuity lasting a year.	Ganjawala et al., 2019
CoChR-GFP	Retinal ganglion cells and Bipolar cells	CAG and mGluR6	Intravitreal	• Triple knockout (TKO) mice model • P30	➢ Substantial pupil constriction was found in RGC-expressing TKO mice. ➢ Restoration in OMR was observed when both RGC and BC are targeted. ➢ RGC expression is much more sensitive to light than BC.	Lu et al., 2020
eNpHR	Cones	• hRHO • hRO • mCAR	None (transgenic mice)	• Mice (rd1) and double-knockout mice • P53–P264	➢ ON, OFF, and ON/OFF light responses at the RGC level were observed, together with lateral inhibition and directional selectivity. ➢ Following treatment, they were able to exhibit optomotor reflexes and light avoidance behavior.	Busskamp et al., 2010
ex3mV1	Retinal neurons	CAG	Intravitreal	• Rat (RCS) • P210–P300	➢ VEP was detected 2 months after injection, indicating that the restoration of visual function was sustained for a significant amount of time.	Watanabe et al., 2021
mGluR6/OPN4 chimera	ON Bipolar cells (rods and cones)	GRM6- SV40/WPRE	Both intravitreal and subretinal	• Mice (rd1) • P >168	➢ V1 activation was greater in rd1-Opto-mGluR6 mice. ➢ In all behavioral tasks, injected *rd1* mice significantly outperformed non-transgenic mice.	van Wyk et al., 2015
mVChR1	Retinal cells	CAG	Intravitreal	Rat (RCS); P180	➢ Treated rats showed functional rescue in behavior that lasted at least a year.	Tomita et al., 2014
MW-opsin	Retinal ganglion cells	hSyn1	Intravitreal	Mice (rd1); P45–60	➢ 1,000-fold more light-sensitive as compared to ChR2, had faster response kinetics, and enhanced contrast sensitivity with increasing levels of contrast, increased VEP amplitude consistent with an increase in firing activity with stimulus onset. ➢ Post-treatment mice were able to discriminate between the light and dark box, detect a spatial light pattern and recognize patterns at different light intensities.	Berry et al., 2019
MCO1 (multicharateristic opsin)	ON bipolar cells (rods and cones)	mGluR6 promoter	Intravitreal	• Mice (rd10) • P60	➢ Increased light sensitivity was observed in visually guided behavior. Protein expression was stable for 4 months.	Batabyal et al., 2021
OPN4	Retinal cells	mCMV	Subretinal	• Mice (rd1) • P30	➢ Increase in the P1 wave of flash VEP lasting only 45 days post injection. ➢ Treated mice spent more time in the dark zone similar to WT	Liu et al., 2016
OPN4	Retinal ganglion cells	CMV	Intravitreal	• Mice (rd1) • P30	➢ Improved visual function following OPN4 expression. ➢ Sensitivity persisted for 11 months.	Lin et al., 2008
OPN4	Retinal ganglion cells	CBA	Subretinal	• Mice (rd1) • P42–45	➢ Mice were able to encode visual information with respect to the background environment. ➢ Improved performance in a behavioral light avoidance assay.	De Silva et al., 2017
ReaChR	Retinal ganglion cells	hSyn1	Intravitreal	• Mice (rd1) • P28–36	➢ Robust responses were evoked in the light avoidance behavioral task.	Sengupta et al., 2016
ReaChR	Retinal ganglion cells	hSyn	Intravitreal	• Mice (rd1) • P28–P35 macaque	➢ Optogenetic activation of targeted cells showed well-defined receptive fields. The firing rate of the neurons showed clear changes to a checkerboard stimulus. This was helpful to design a model giving a strong estimation to determine visual acuity after optogenetic vision restoration.	Ferrari et al., 2020
Rho and ChR2 (H134R)	ON Bipolar cells	4xgrm6	Intravitreal	• Mice (rd1) • P21–42	➢ Stable (green light) responses with an amplitude similar to WT were observed in a wide range of light intensities. Response kinetics slower. ➢ Mice were able to distinguish between light and dark as well as static and moving spatial light patterns.	Gaub et al., 2015
Rho	ON Bipolar cells	grm6 and CAG	Intravitreal	• Mice (rd1) • P56–70	➢ Responses from dLGN had variable amplitude and latency, which were either sustained or transient in nature. ➢ Treated mice could recognize changes in luminance, flickers of different frequencies, spatial patterns, and natural movie scenes.	Cehajic-Kapetanovic et al., 2015

ChR2, channel rhodopsin; Rd, Retinal degeneration; VEP, visually evoked potential; Rho, rhodopsin; OPN4, melanopsin; ReaChR, red-shifted channel rhodopsin; mVChR1, modified version of channel rhodopsin; MW opsin, medium wavelength cone opsin; CAG, chicken beta-actin promoter; CMV, cytomegalovirus; PLR, pupillary light reflex; GRM6, glutamate metabotropic receptor; hRHO, human rhodopsin hRO, human red opsin; mCAR, mouse cone arrestin-3; WPRE, woodchuck hepatitis post-transcriptional regulatory element; SV40, Simian vacuolating virus 40; mGluR6, metabotropic glutamate receptor. RCS, Royal College of Surgeons; eNpHR, natronomonas halo rhodopsin; hSyn, human synapsin; WT, wildtype; Chop-2, channelopsin; CBA, hybrid CMV enhancer/chicken beta-actin; Thy-1, thymocyte differentiation antigen 1; SNCG, gamma synuclein; TKO, transgenic knockout mice; Co-ChR, *chloromonas oogama*.

## Visual responses with expression in bipolar cells

During RD, the cell loss is mostly confined to the outer retinal photoreceptors while the BCs and RGCs are spared and remain intact even at the end stage (Bi et al., 2006). Targeting the BCs offers the advantage of providing natural signaling via inherent retinal circuitry and demonstrates reliable, stable cortical responses upon treatment. The signal convergence from bipolar cells to retinal ganglion cells enhances light sensitivity due to their potential for photon catch. These upstream cells have the advantage of not being affected by significant remodeling at end-stage RD (Gaub et al., 2015). A summary of the studies is given in Table 1.

Initial attempts to preserve inner retinal signaling were made by Lagali et al., where ChR2 was introduced in the ON-bipolar cells (both rod and cone) of the retina via electroporation in neonatal rd1 mice (Lagali et al., 2008). To determine if the photo responses were transmitted downstream to the cortex, the visually evoked potentials (VEPs) were recorded from the primary visual cortex (V1). The amplitude of the VEP responses from the treated mice was significantly larger as compared to untreated blind mice. Interestingly, the shape of the VEP was different from the WT mice which could be due to the difference in the retinal output as one eye of the mice was electroporated. With the groundbreaking demonstration of light sensitivity achieved at the cortical level by expression of a genetically encoded neuromodulator in different subtypes of ON-bipolar cells, successive studies were carried out to check the efficacy at late-stage RD (Doroudchi et al., 2011). Targeted expression in the ON bipolar cells (rod and cone) with ChR2 transgene led to robust expression that lasted almost 10 months post-injection. The long-lasting expression of the transgene enabled the treated mice for improved behavioral performance in a visually guided behavior. The mice did not perform well at lower light intensities but improved remarkably with increments in light intensity that was close to wild-type (WT) mice (Doroudchi et al., 2011). Furthermore, Mace et al. developed an ON-bipolar cell-specific promoter with a novel AAV vector, 7m8 that had improved transduction efficiency (both cone and rod) compared to AAV2 parental serotype used in previous studies (Macé et al., 2015). The recovery of visual responses was measured by the peak amplitude of the visually evoked potentials (VEPs) and multiunit activity (MUA) from V1. The VEP amplitude was lower but had a similar shape as compared to wild-type mice. Spikes were elicited in the cortex in response to both light increments and decrements across several repetitions, and the latency of ON responses was shorter, whereas the OFF responses were similar in both treated and WT mice. The difference in latency for ON responses can be attributed to direct synaptic transmission by cone bipolar cells to RGCs and further downstream the visual pathway. The other plausible reason could be the amplified signals originating from rod bipolar cells activating several cone bipolar cells. The observed OFF responses may result from the activation of AII amacrine cells by the rod ON bipolar cells that have glycinergic synapses with OFF bipolar cells. The physiological responses of the cortex were consistent with a light avoidance task where the mice showed quick locomotory behavior in response to the light stimulus. This study demonstrated that optogenetic treatment of bipolar cells retains the ability to transmit visual information even after full photoreceptor degeneration. Despite favorable results with microbial opsins at the cortical and behavioral level, they have the disadvantage of requiring higher intensities of light, while native opsins can function with lower intensities of light possibly due to signal amplification cascades, and pose less risks to the immune response. The selective expression of human rod opsin in the ON-bipolar cells (cone and rod) has been shown to effectively restore vision in blind mice (Cehajic-Kapetanovic et al., 2015). Electrophysiological recordings from retinal explants and the visual thalamus exhibited changes in firing (increase and decrease) induced by simple light pulses, luminance change, and naturalistic movies in treated mice. The overall responses were of mixed type with variability in their amplitude, latency, and duration of responses. The animals demonstrated an improved ability to discriminate between light increment and decrement in a visual discrimination task, and detect flicker and gratings of different frequencies and contrast. In addition, there was an increase in visual responses to natural movies that involved changes in elements of natural scene. A further addition to the already existing optogenetic toolkit was a newly engineered melanopsin-glutamate receptor chimera, Opto-mGluR6 (van Wyk et al., 2015). Melanopsin is a blue light-sensitive photopigment responsible for the pupillary light reflex and circadian rhythm. It resides in a small population of retinal ganglion cells called intrinsically photosensitive retinal ganglion cells (ipRGCs) (Hattar et al., 2002). The modified G protein-coupled receptor (GPCR) consists of a transmembrane domain of melanopsin and an intracellular domain of ON-bipolar cell-specific mGluR6. The transduction of this chimeric gene in the retina (both rod and cone) of *rd1* mice showed elevated activity in response to visual stimulus. The animal had better visual performance in a visually guided swim task after a few days of training. Learning was not affected even at subthreshold light intensities as the animal performed equally well in this behavioral task, demonstrating high sensitivity of this protein (van Wyk et al., 2015). Melanopsin-based chimeras expressed in both rod and cone ON bipolar cells have been demonstrated to restore cortical light responses (Kralik et al., 2022) but have a higher latency of evoked response unlike MW opsin (Berry et al., 2019) and do not elicit OFF responses different from previous studies (Macé et al., 2015).

Overall, targeting the bipolar cells with microbial opsins showed VEPs with different shapes and higher amplitude compared to WT mice. On the other hand, bipolar cells expressing vertebrate opsins had smaller amplitude VEPs exhibiting ON and sometimes OFF responses. Expression of chimeric constructs in the bipolar cells led to improved light sensitivity attributed to intracellular signal amplification by the G-protein metabotropic signal cascade. The inconsistency of OFF responses could arise from a compromised rod bipolar pathway. The observed variability in the responses could result from different contributing factors such as the method of vector delivery, age of the mice, efficiency in transducing bipolar cells, and non-specific labeling of cells (amacrine and RGCs). Until now, the focus had been to target only the ON-bipolar cells (rod and cone), and the OFF pathway has not been explored so far. Simultaneous targeting of both the cell types would help to attain better visual perception.

## Visual responses with expression in retinal ganglion cells

The RGCs are the output neurons of the retina responsible for transmitting visual information to the cortex. The visual capability upon transduction of a transgene in RGCs has been investigated by several research groups. ChR2-YFP expressing in roughly 30% of RGCs in blind mice (ChR2*rd1/rd1*) could discriminate between bright and dark stimuli with no measurable cortical activation even with high-intensity stimulus similar to *rd1/rd1* mice (Thyagarajan et al., 2010). The lack of visible responses from the cortex could arise from the restricted expression only in the ganglion cell class that project to the superior colliculus and not to the downstream visual structures. The transduction of ChR2 in RGCs of aged dystrophic RCS rats (*rdy/rdy*) was evaluated for visual function (Tomita et al., 2010). The amplitude of the VEPs in rats increased with an increase in light intensities in comparison to dystrophic rats where no responses were observed. The latency was shorter as the transduced signals in the RGCs were transmitted directly to the cortex and not through the inner retinal network. The optomotor responses of the treated rat also showed better responses with increasing light intensities. Since ChR2 requires high light intensities and stimulation with blue light increases the risk of inducing photochemical damage in the retina, better alternatives were devised to use red-shifted channel rhodopsin variants (ReaChR) by increasing the range of light intensity (Lin et al., 2013; Sengupta et al., 2016). ReaChR-treated mice were stimulated with light intensities below the safety limit threshold of the human eye. The cortex was activated by light ON but not to light OFF as all RGCs turned into ON cells and the ON-OFF pathway could not be restored. Another attempt to deal with the problem of potential cell damage was to use a ChR2 mutant that has a higher permeability for Ca^2+^ ions, named CatCh (Kleinlogel et al., 2011). The protein is 70 times more light-sensitive than ChR2 and can generate fast action potentials with lower light intensity. Selective targeting of this protein in the RGCs of *rd1* mice was evaluated for cortical responses to increasing light intensities. The treated blind mice responded to lower light intensities (10^15^ photons/cm^2^/s), with short latency compared to the ON responses of wild-type mice. CatCh had a higher transduction efficiency in the peri-foveolar RGCs in macaques exhibiting robust-spiking activity from a population of RGCs with light intensities from 10^14^ photons/cm^2^/s intensity and above (Chaffiol et al., 2017). More variants of ChR2, CoChR (*Chloromonas oogama*) were developed in terms of improved light sensitivity by optimizing the kinetics such as an increase in deactivation time or off rate (Ganjawala et al., 2019). The efficacy of this variant was tested in triple knock-out transgenic blind mice via a multielectrode array (MEA) recordings and optomotor behavioral assays. The threshold light intensity for RGC response was almost 2 log units lower than ChR2 and ReaChR (1.9 × 10^12^ photons/cm^2^/s). This was consistent with optomotor responses elicited at ambient light intensities similar to the LCD monitor.

With the proof of concept established with an expression of ChR2 in RGCs, researchers explored the potential of vertebrate opsins such as melanopsin and cone opsins as new biological prosthetics in restoring vision as modification of existing ones. In a previous study, the expression of mouse melanopsin in the ganglion cells rendered them intrinsically light responsive and showed uniform responses driven by melanopsin (Lin et al., 2008). These mice performed well in a two-choice visual discrimination task. The pupillary light reflex was stable and lasted until 11 months of injection showing the capacity of ectopic expression of melanopsin protein in mediating visually driven information. Subsequently, the outcome of the human melanopsin gene (OPN4) was tested for long-term vision restoration at later stages of RD in blind mice (De Silva et al., 2017). Subretinal injection of the construct led to widespread expression in the retina and increased blood flow induced by light stimulation. The injected mice showed improved performance in an object recognition task and the effects lasted 13 months after injection.

A recent strategy to endow light sensitivity was by using cone opsins, middle wave opsin (MW-opsin) which are G-protein-coupled receptors that are very sensitive to light. The goal was to achieve faster kinetics, sensitivity, and better adaptation to ambient light (Berry et al., 2019). The *rd1* mice expressing the MW-opsin showed a strong preference for staying in the dark compartment in a light avoidance task. Furthermore, these mice could discriminate between constant and flashing light, distinguish moving lines of different spatial frequencies, and explore novel objects over a wide range of natural light conditions. Electrophysiology experiments from the V1 showed better detection of stimulus with changes in response to changes in brightness. With notable achievements obtained from the above methods, researchers are now trying for preclinical validation in primates. In non-human primates, the spatial resolution of the retina has been estimated to determine the visual acuity treated by optogenetic therapy (Ferrari et al., 2020). The size of the receptive field of ganglion cells was measured in monkeys injected with optogenetic proteins. Most cells had a smaller receptive field indicating the whole neuron of the axon not being light-sensitive after reactivation. The ganglion cells are sensitive to stimulation of only their axon and dendritic field. The finding is promising and suggests optogenetic therapy as a better strategy compared to retinal implants where electrical stimulation can activate distant ganglion cells thereby limiting visual acuity. In *rd10* mice whose visual responses decline at an advanced age (P99), upon treatment with a red-shifted channelrhodopsin, ChrimsonR showed robust, long-lasting responses to UV light and red light stimulation (Cheong et al., 2018). This was due to the expression of the protein lasting for an extended time in the ganglion cells. The therapeutic efficacy of ChrimsonR in efficient signal transmission to the higher visual areas of the brain has been demonstrated in primates as well (Gauvain et al., 2021; Chaffiol et al., 2022; McGregor et al., 2022). In primates, the responses compared before and after stimulation using synaptic blockers (block glutamatergic transmission of the retina) allowed the selected stimulation of transduced cells and not the entire retinal pathway. The evoked potentials followed the train of pulses of specific frequencies more frequently after blocker administration. Responses were fast, and the amplitude increased with the increase in light intensity. The protein expression lasted ~20 months after injection in the perifoveal region of the eye. In a recent clinical study, Chrimson R was targeted to foveal retinal ganglion cells into the worse-seeing eye of a patient suffering from retinitis pigmentosa (RP). The therapy was combined with specialized light-stimulating goggles (Sahel et al., 2021). The patient was trained 4.5 months after injection, allowing the stabilization of the expression of the transduced gene. After 7 months of training, the patient started to show signs of visual improvement in a visual detection task that enabled him to perceive, locate, and touch different objects with the help of goggles. The quality of vision restored cannot be compared with natural vision since there are multiple subtypes of RGCs that encode diverse features and send highly processed images to the brain. Further research into developing tools targeting more varied cell types of RGCs would help to restore more natural vision.

The functional outcome of differential targeting of the BCs and RGCs of AAV-mediated optogenetic gene therapy was compared in a triple knock-out mouse model (Lu et al., 2020). Electrophysiological recordings from the retina showed RGCs to have a light intensity (2.0 × 10^13^ photons/cm^2^/s) that was approximately a log unit lower than that of BCs (2.4 × 10^14^ photons/cm^2^/s). Better performance with RGC transfection was also observed with optomotor responses displaying higher sensitivity with a lower threshold of light intensity. In a recent study, both mammalian (human melanopsin, hOPN4) and microbial opsin (ReaChR) were targeted to the RGCs and bipolar cells to compare their kinetics and sensitivity (Gilhooley et al., 2022). As compared to RGCs, targeting ON bipolar cells with hOPN4 exhibited higher light sensitivity being able to encode a range of light intensities and rapid decay kinetics. ReaChR had a limited dynamic range of encoding, but it exhibited faster decay kinetics selectively only in ON bipolar cells. Until now, there is no common consensus on the preferred cell type for targeting as each of them offers elements of advantages and disadvantages, and further comparative studies are necessary to determine the best choice. A summary of the above studies is given in Table 1.

## Conclusion

Optogenetic strategies for vision restoration have gained impetus using a wide variety of transgene and vector combinations. The success of this approach has been through the continuous process of improving the transduction mechanism and cell-specific expression profile of the optogene. The initial studies that started with ChR2 gradually expanded to a safer and broader spectrum in addition to the G protein signaling cascade for better temporal resolution. With the proof of principle demonstrated by superior spatial and temporal visual resolution in animals and primates, these tools are utilized in clinical trials as viable treatment for inherited retinal degeneration. The outcome has been promising as the patients have shown partial functional recovery being able to recognize the pattern and presence of large objects. However, there are several factors that have limited the outcome of gene therapy. One such challenge is the selective targeting of the retinal cell types in healthy primates and degenerated human retina and its potential immune response with microbial opsins. Until now, there are no data about the performance of vertebrate opsins in primates or humans. Further development is required in designing promoters and engineering opsins will enable reliable targeting of different cell types yielding increased light sensitivity and better temporal kinetics. Next, extensive inner retinal rewiring at advanced stages of RD leads to the emergence of rhythmic activity and oscillations of the local field potentials in the retina that further affects the central visual circuitry (Biswas et al., 2014; Goo et al., 2015; Kalloniatis et al., 2016). Currently, there is a lack of information on whether early- or late-stage degeneration yields the best therapeutic result. This necessitates more functional studies to understand the implications of RD on the response properties of the central visual circuitry at different stages of degeneration. Recent studies are therefore trying to determine the proper stage of intervention to achieve the maximal benefits of optogenetics in obtaining complete visual restoration.

## Author contributions

AB contributed to conception, design of the study, and wrote the manuscript. AB and KP did literature collection and made the figures and tables. Both authors approved the submitted version.
